# iDS372, a Phenotypically Reconciled Model for the Metabolism of *Streptococcus pneumoniae* Strain R6

**DOI:** 10.3389/fmicb.2019.01283

**Published:** 2019-06-25

**Authors:** Oscar Dias, João Saraiva, Cristiana Faria, Mario Ramirez, Francisco Pinto, Isabel Rocha

**Affiliations:** ^1^Centre of Biological Engineering, University of Minho, Braga, Portugal; ^2^Instituto de Microbiologia, Instituto de Medicina Molecular, Faculdade de Medicina, Universidade de Lisboa, Lisbon, Portugal; ^3^BioISI – Biosystems & Integrative Sciences Institute, Faculdade de Ciências, Universidade de Lisboa, Lisbon, Portugal; ^4^Instituto de Tecnologia Química e Biológica António Xavier, Universidade Nova de Lisboa (ITQB-NOVA), Oeiras, Portugal

**Keywords:** genome-scale metabolic model, *Streptococcus pneumoniae* R6, metabolic reconstruction, iDS372, avirulent, phenotypical reconciliation

## Abstract

A high-quality GSM model for *Streptococcus pneumoniae* R6 model strain (iDS372), comprising 372 genes and 529 reactions, was developed. The construction of this model involved performing a genome-wide reannotation to identify the metabolic capacity of the bacterium. A reaction representing the abstraction of the biomass composition was reconciled from several studies reported in the literature and previous models, and included in the model. The final model comprises two compartments and manifold automatically generated gene rules. The validation was performed with experimental data from recent studies, regarding the usability of carbon sources, the effect of the presence of oxygen, and the requirement of amino acids for growth. This model can be used to better understand the metabolism of this major pathogen, provide clues regarding new drug targets, and eventually design strategies for fighting infections by these bacteria.

## Introduction

The number of studies in the field of genomics has significantly increased with the rise of new next-generation sequencing techniques. A sequenced genome allows the reconstruction of genome-scale metabolic (*GSM*) models (Dias et al., 2015), providing insights into the metabolism of an organism of interest.

Genome-scale metabolic models have been increasingly used as bioinformatics tools for the analysis of metabolism, either for the identification of potential target sites (Igoillo-Esteve et al., 2007) or over-production of compounds of interest (Zhang et al., 2006; Lee et al., 2009). In a general sense, *GSM* models provide insights into metabolic conversions based on genomic information and allow the analysis of metabolic pathways.

The development of *GSM* models has been described in detail elsewhere (Thiele and Palsson, 2010; Dias and Rocha, 2015) and several tools were developed to automate this process, such as *merlin* (Dias et al., 2015) and others (Hamilton and Reed, 2014). The reconstruction process usually involves four steps, namely the genome annotation, the assembly of the reactions network, the conversion of the network to a model, and the validation of the model with biological data from previously published or specifically designed experiments.

However, the scarcity of literature and biological data remains an obstacle in reconstructing *GSM* models for organisms whose metabolic capacity is poorly characterized. Organisms such as *Escherichia coli*, for which large amounts of biological and experimental data that greatly facilitate the reconstruction process are accessible, have already several models available (Edwards and Palsson, 2000; Reed et al., 2003; Feist et al., 2007; Orth et al., 2011).

*Streptococcus pneumoniae* (pneumococcus) is a Gram-positive, lactic acid bacterium, which not only asymptomatically colonizes the nasopharynx of humans, particularly of young children, but is also a major human pathogen, responsible for diseases such as otitis media, pneumonia, or meningitis. The *S. pneumoniae* R6 strain is one of the best studied strains in this species. It is a non-capsulated, highly competent derivative of a serotype 2 strain isolated from a child in the early 20th century. There is still a limited amount of biological information and literature available for this microorganism, although relatively recent studies have focused in reducing this gap (Carvalho, 2012; Härtel et al., 2012; Hathaway et al., 2012; A. Nieto et al., 2013). Moreover, there is significant resistance to the antimicrobials of choice for treating these infections (Jothi et al., 2008; Ding et al., 2009; McAllister et al., 2012). A better understanding of its metabolism is essential in providing clues to new drug targets (Sham et al., 2012) as well as for understanding the transition between colonization and disease and the adaptations to survive in the various sites pneumococci can occupy and invade in its human host.

Based on genomic information, strain R6 lacks genes encoding for the enzymes of the Entner–Doudoroff pathway, Krebs cycle, and any proton chain reaction for either aerobic or anaerobic respiration (Härtel et al., 2012). Therefore, these bacteria present a fermentative metabolism, independently of the presence of oxygen (Hoskins et al., 2001). However, the fermentative profile in an aerobic environment, in this organism, switches from lactate to acetate as a major by-product, boosting growth in 37% when compared to anaerobic conditions (Carvalho et al., 2013). The metabolic changes that contribute to this behavior include inactivation of the *pfl* gene, the activation of pyruvate oxidase (SpxB) which contributes to the formation of H_2_O_2_ and acetate from pyruvate and also the expression of flavin-type NADH oxidases that reduce O_2_ to less toxic forms (e.g., NOX gene) (Carvalho, 2012). Nevertheless, strain R6 is a catalase-negative organism and uncapable of leading with the toxicity of this oxidative metabolism for a long period of time.

The purpose of this work was constructing a high-quality *GSM* model for *S. pneumoniae* strain R6, to perform comprehensive comparative studies between experimentally determined and computationally predicted phenotypes, under different environmental conditions and with various genetic alterations. This model will allow gaining insights into *S. pneumoniae* physiology and metabolism, beyond what the experimental data has been providing.

## Materials and Methods

### Online Databases

Several databases were used throughout this work to aid in all stages of the study. A brief description of the information retrieved from each database is available in Supplementary Table S1.

### Genome Sequence

The genome sequence with the NCBI assembly accession number ASM704v1 was retrieved from the GenBank repository.

### Merlin

The development of the *GSM* was supported by *merlin* (Dias et al., 2015, 2016, 2018). This platform allows performing several steps of the reconstruction process semi-automatically, while providing user-friendly graphical user interfaces for reviewing information and performing manual curation. Below a detailed description of the main procedures is provided.

### Metabolic Model Reconstruction

**FIGURE 1:**
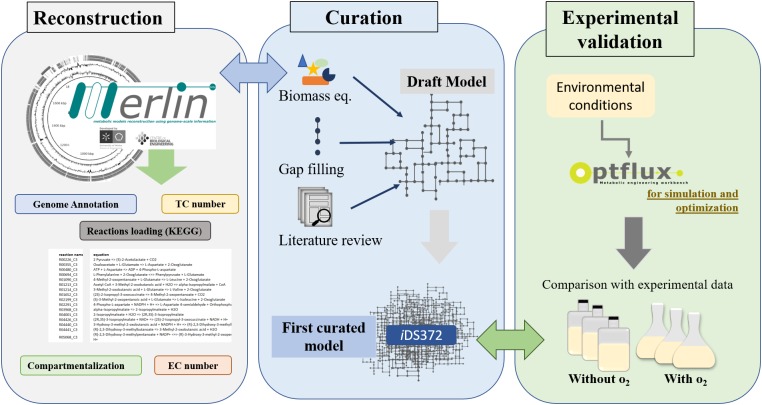
Workflow for metabolic network reconstruction of *S. pneumoniae* R6 (EC, Enzyme Commission Number; TC, Transporter Classification). A draft of the network is reconstructed semi-automatically by *merlin*. The genome re-annotation, compartmentalization, and manual curation are performed using this user-friendly’s graphical user interfaces. Next, the biomass equation is formulated, resorting to available experimental data for *S. pneumoniae* R6 or closely related organisms (as determined via 16s rRNA analysis). Additionally, literature is also analyzed to improve the biomass equation. Environmental conditions, to mimic experimental data, are defined next. Using the software Optflux, simulations are performed using the model under the previously established environmental conditions. The simulated growth is compared to experimental data. If the results are distinct then the model is further reviewed and curated and the process is repeated until no significant differences exist between the experimental results and those obtained *in silico*.

The workflow for the reconstruction process is shown in Figure 1, encompassing the main steps described in the section “Introduction”.

#### Genome Annotation

##### Phylogenetic tree

The identification of homologous genes is most likely to occur in closely related organisms (Edwards et al., 2002). Although markers are available to distinguish *S. pneumoniae* species (Scholz et al., 2012), phylogenetic tree also provides a form of establishing proximity between species. For that purpose, the 16S rRNA gene of several species (Lane et al., 1985) was used in this assessment. The sequences of closely related organisms (Supplementary Figure S1) were retrieved from NCBI’s database and aligned using EMBL-EBI Clustal OMEGA multiple sequence alignment tool (Sievers et al., 2011) to produce the phylogenetic tree^1^.

##### Semi-automated genome annotation

*Merlin* (Dias et al., 2015) software allows performing the genome functional annotation, assigning scores based on taxonomy and frequency of similar sequences, to assign enzyme commission numbers (EC number) and enzymatic functions. These EC numbers are then used to select which Kyoto Encyclopedia of Genes and Genomes (KEGG) (Kanehisa et al., 2004) reactions are going to be included in the model. Two tools, Basic Local Alignment Search Tool (BLAST) (Altschul et al., 1990) and HMMER (Finn et al., 2011), were used within *merlin* to perform similarity searches.

##### Re-annotation workflow

New gene functions assignments and corrections may have a significant impact in model performance. Therefore, genome-wide re-annotations should be carried out periodically, to retrieve the most up-to-date genomic information. For this purpose, a re-annotation workflow was developed and implemented, as shown in Supplementary Figure S2, and annotation labels were assigned to each gene’s annotation proposed by *merlin*. The assignment of labels (from A to E) makes the annotation traceable, as each leaf of the workflow indicates a different decision regarding each gene’s annotation. Moreover, the labels can also be seen as an indicator of the confidence in a particular annotation.

The analysis relied on the assignment of EC numbers to each enzyme or putative enzyme encoded in the genome. For the cases in which a complete EC number had been identified by *merlin*, candidate metabolic genes were analyzed by verifying if an EC number was also identified in UniProt for that specific protein either in *S. pneumoniae* R6 or other *S. pneumoniae* strains. If true, an additional analysis was performed to verify if *merlin*’s classification matched the one in the UniProt database. Label A was assigned to genes encoding proteins with matching functions in UniProtKB/Swiss-Prot and label B to genes encoding proteins with matching functions in UniProtKB/TrEMBL.

When there were conflicting EC numbers, priority was given to proteins in UniProt/Swiss-Prot when assigning gene functions (Label C). If no EC number was present in UniProt, then the existence of multiple complete EC numbers, identified by *merlin* during genome annotation, was evaluated. If only one complete EC number with a classification score >0.2 (threshold empirically determined after analysis of the results) was available, then the annotation was accepted. In cases where more than one complete EC number was present, priority was given to those that matched the homologous gene of another reference strain (Label D). *S. pneumoniae* strain D39 was selected as the first reference strain, while *Lactococcus lactis* strain NZ9000 was the alternative reference if no homologous genes existed in D39. For cases in which no matching gene function was ascertained from the reference organism, additional tasks, such as BLAST searching against proteins in UniProt/Swiss-Prot, were carried out in a final effort to identify a gene function. These annotations are identified with Label G.

If an incomplete EC number was identified, an alternative complete one was sought in *merlin*. If available, the re-annotation was carried out in the same manner as in the cases in which no EC number was present on UniProt and therefore the gene was labeled G, F, or discarded, according to the workflow shown in Supplementary Figure S2.

In case of an absent complete EC number, a search across multiple databases such as BRENDA (Scheer et al., 2011), UniProt, Conserved Domain Database (CDD) (Marchler-Bauer et al., 2013), BioCyc (Karp et al., 2005), and KEGG as well as in literature was carried out. If enough information to support the assignment of a gene function was found, then it was added to the model with Label E.

All cases that did not meet the minimum requirements stated in the re-annotation workflow were discarded from the model.

Note that the presence of an EC number after annotation does not necessarily mean that the gene will be included in the metabolic model. Such are the cases of genes involved in DNA and RNA processes such as methylation or rRNA modification, as well as pseudo-genes among others.

#### Assembling the Metabolic Network

The assembly of the metabolic network involves collecting a set of reactions. In *merlin* these reactions are retrieved from KEGG. Such reactions should be spontaneous or promoted by enzymes encoded in the organism’s genome. Hence, the genome annotation determines which reactions will be included in the model. The algorithm used by *merlin* for the assembly of the metabolic network from the annotated genome is described in detail elsewhere (Dias et al., 2015).

The stoichiometry of reactions in the network should be verified to guarantee that all reactions are balanced. Likewise, the reversibility of the reactions should also be confirmed to avoid gaps and mispredictions of the model. The reversibility may be determined from the estimation of the standard Gibbs free energy of formation (Δ*f* G′0) and of reaction (Δ*r* G′0), by analyzing manually curated *GSM* models of closely related organisms or by biochemical studies of the enzymes.

*Merlin* includes tools to determine the balance of the reactions, as well as information on reversibility retrieved from a study by Stelzer et al. (2011) that analyzed reactions available on the KEGG database. Finally, reactions labeled as unbalanced by *merlin* as well as the direction of reactions set as irreversible were manually verified.

##### Compartmentalization

The compartmentalization of the model is based on results obtained from PSORTb 3.0 (Nakai and Horton, 1999; Yu et al., 2010).

##### Transport reactions

The compartmentation of the model leads to the need for defining carriers between compartments. The Transporter Classification Database (TCDB) is a repository of transport protein encoding genes, reported in the literature. *Merlin* uses TRIAGE (Dias et al., 2017) for retrieving information from TCDB to create an internal curated database. TRIAGE was used to identify carriers in the studied genome by determining which genes have transmembrane domains, similarities to TCDB and are known to be in the membrane. Finally, TRIAGE generated specific transport reactions associated with those genes, and added them to the model.

##### Genes, proteins, and reactions

The genes–proteins–reactions (GPR) associations are usually determined by searching biological databases and literature. However, determining if the genes encode subunits of a single protein, isoenzymes, or different proteins belonging to a protein complex may not be straightforward. Hence, *merlin* uses information retrieved from KEGG BRITE to implement these rules.

*Merlin* searches for the structure of the protein complex modules, including their subunits, and the stoichiometry of every EC number available in the model. Subsequently, this tool parses the data, identifying the orthologs required by each GPR rule and searches for these sequences in the studied organism’s genome, thus identifying the rule and the subunits of the protein in the model (Dias et al., 2015, 2018).

#### Converting the Metabolic Network to a Stoichiometric Model

##### Biomass equation

The biomass equation aims to account for all compounds that compose the cellular biomass. In the absence of experimental data to support the definition of the biomass reaction, one must rely on information obtained through its genome composition and from closely related organisms. This step is essential, considering that the lack of biomass precursors might affect validation procedures. If a potentially essential precursor is not included in the biomass equation, then reactions that lead to its production and, consequently, the corresponding protein encoding gene(s) are rendered non-essential.

The *L. lactis* (iAO358) model (Oliveira et al., 2005) was used as a template for the overall macromolecular biomass composition. Since *S. pneumoniae* strain R6 does not have a capsule, the polysaccharide macromolecule present in iAO358 was excluded and new coefficients were calculated, maintaining the relative abundance.

While the composition of the lipid macromolecule, in terms of which molecules are required to assemble the lipid, was also inferred from iAO358, the subcomponents of other macromolecules and several of their precursors were retrieved from the literature, namely the composition of the average fatty acid (Behr et al., 1992), peptidoglycan (Mosser and Tomasz, 1970; Delcour et al., 1999), teichoic acid (Mosser and Tomasz, 1970; Fischer, 1997; Delcour et al., 1999), and lipoteichoic acid (Behr et al., 1992; Delcour et al., 1999; Draing et al., 2006). Essential cofactors were determined according to the work of Xavier et al. (2017) and conditional cofactors from previous studies (Shah et al., 2011; Potter et al., 2012). The remaining components were determined from genome information, namely the amino acid, nucleotide, and deoxynucleotide contents. For this, a bioinformatics tool (e-BiomassX) developed in-house and available in *merlin* (Santos, 2013) was employed. This tool implements a strategy similar to the one reported previously (Thiele and Palsson, 2010). The protocol of Thiele and Palsson (2010) does not take into account that cells contain different types of RNA and uses only mRNA to determine RNA contents. In this approach, three types of RNA were used: mRNA, tRNA, and rRNA in the proportion of 5, 20, and 75%, respectively (Neidhardt, 1996).

##### Growth and maintenance ATP requirements

The growth and maintenance ATP requirements were calculated with data retrieved from previous work (Carvalho et al., 2013). In anaerobic conditions, the ATP yield reported in the above-mentioned study is 2 mol ${\text{mol}}_{\text{Glc}}^{-1}$. Hence, by multiplying this yield by the specific rate of substrate uptake (*q*_S_), the growth ATP requirements are obtained. Although these requirements should be adjusted to different growth conditions, it was assumed, due to lack of additional data, that growth ATP requirements were the same for all genetic and environmental conditions, which is a common practice in *GSM* models. Therefore, this value was used for all simulations presented in this work. Likewise, 1 mmol.g_bio_^−1^ h^−1^ was used as the initial flux value (FVA) for maintenance ATP. However, this flux was adjusted to experimental data, both for anaerobic and aerobic conditions, as described in the section “Results”.

#### Validation of the Metabolic Model

The evaluation of the simulation performance under physiologically meaningful environmental conditions is essential for the validation of *GSM* models. Whenever experimental data are available, a comparison between simulations and experiments is easily performed, whereas when such information is not available model accuracy can be impaired. For this work, physiological data detailed in available references were used.

##### Aerobic and anaerobic metabolism

Carvalho (2012) compared strain R6 and D39 in different defined media and environmental conditions. According to this study, in strain R6, 1% (w/v) of glucose concentration in the medium promotes a faster growth, while higher concentrations have an inhibitory effect on the specific growth rate (from 0.69 h^−1^ at 1% to 0.47 h^−1^ at 3% of glucose), originating also a prolonged lag-phase and lower biomass production. Furthermore, growth and fermentation profiles were measured in semi-aerobic, anaerobic, and aerobic conditions, under pH controlled at 6.5. In semi-aerobic conditions, R6 grows until glucose is depleted from the medium (1% w/v) producing mainly lactate and minor amounts of acetate, formate, and ethanol. Under anaerobic conditions, strain R6 specific growth rate slightly increases (0.69 h^−1^ to 0.78 h^−1^) and by-products formation exhibits the same profile observed in semi-aerobic environment. The lower growth rate observed for semi-aerobic conditions can be explained by the higher pyruvate oxidase activity this strain exhibits, when compared with strain D39, whose enzymatic reaction has H_2_O_2_ as by-product, which is notably known to arrest growth in *S. pneumoniae*. Fully aerobic conditions drastically change growth and by-products accumulation in R6. For instance, specific growth rate increases to 1.07 h^−1^ for a short period of time (2 h), which entails a 16-fold decrease in biomass yield regarding semi-aerobic conditions. Metabolism shifts from lactate to acetate and H_2_O_2_ production, denoting also a high activity of pyruvate oxidase. Also, formate is no longer produced, revealing a total inhibition of pyruvate formate lyase (PFL). Upon growth arrest, lactate is consumed to produce acetate and H_2_O_2_, with generation of ATP. However, due to the impossibility of determining oxygen consumption rate in semi-aerobic conditions, simulations will only be performed for studies in anaerobic and aerobic conditions, henceforth referred to as study 1 and study 2, respectively.

##### Metabolism with different carbon sources

Due to lack of information regarding carbon sources other than glucose for strain R6, the results published for parental strain D39 were also considered in this work. Paixão et al. (2015b) characterized growth profile, growth rate, and end-products formation in a semi-aerobic environment in chemically defined media (CDM) with glucose, *N*-acetyl-D-glucosamine, mannose, and galactose. These sugars were chosen for their natural presence in the human nasopharynx. As stated above for strain R6, strain D39 produces mainly lactate in the presence of glucose as sole carbon source and in minor quantities acetate and ethanol. Formate is also formed but in values below the limit of quantification. The same profile of full homolactic fermentation is observed for growth in *N*-acetyl-D-glucosamine, although for this carbon source, formate and ethanol are produced in higher quantities. Curiously, the specific growth rate in *N*-acetyl-D-glucosamine decreases to 0.55 h^−1^ in comparison with 0.9 h^−1^ obtained in glucose and reaches a 1.3-fold lower optical density (OD 2.29 vs. OD 1.76). Consumption of mannose also denotes homolactic fermentation, although the products of mixed-acid fermentation increase 2.9-fold for acetate, 5.5-fold for ethanol, and 3-fold for formate in comparison with its production in *N*-acetyl-D-glucosamine. Growth rate and biomass production in this carbon source are similar with fermentation in *N*-acetyl-D-glucosamine. Lastly, strain D39 shows a mixed-acid fermentation profile on galactose, producing formate (in higher quantities), ethanol, and acetate in a proportion of 2:1:1. Lactate is detected as a minor product of fermentation. The specific growth rate for this carbon source is 0.48 h^−1^ and maximal OD is 2.16. This study henceforth referred to as study 3 will encompass the assessment of the model with all four carbon sources.

##### Influence of the availability of exogenous amino acids on growth

Härtel et al. (2012) performed isotopolog experiments to identify amino acid biosynthesis pathways in *S. pneumoniae* strain D39 in chemically defined medium supplemented with glucose. Briefly, one of the experiments performed in Härtel’s study aimed at determining which amino acids were essential for growth. Härtel et al. (2012) performed experiments in which they attempted to grow *S. pneumoniae* D39 in CDM in which each amino acid was separately omitted. Analysis showed that pneumococci are auxotrophic for L-arginine, L-cysteine, L-glutamine, glycine, L-histidine, L-leucine, L-isoleucine, and L-valine. Härtel et al. (2012) were able to identify an unconventional pathway for the *de novo* biosynthesis of serine and have demonstrated the dual utilization of carbohydrates and amino acids by pneumococci. This work henceforth referred to as study 4 will be used to assess the impact of amino acids on organism growth.

##### Gene essentiality

The Online Gene Essentiality database (OGEE) (Chen et al., 2012) was accessed to retrieve experimental data regarding gene essentiality studies for *S. pneumoniae* R6. Additionally, determining the function of genes identified as critical involved assessing information retrieved from several databases (Supplementary Table S1) and literature. The assessment of these data to model prediction henceforth referred to as study 5 will be used to assess the accuracy of the model regarding essential genes.

##### Carbon repression

For strain D39, it has been described previously (Carvalho et al., 2011) that when glucose is used as carbon source, the Catabolite Control Protein A (CcpA) represses genes involved in mixed-acid fermentation, namely *pfl* (spr0415), *pflF* (spr0232), *ackA* (spr1854), *adh* (spr1866), *pta* (spr1007), *lctO* (spr0627), and activates *ldh* (spr1100). When galactose is used as the carbon source, the enzymes downstream of pyruvate exhibit alleviation of repression of the genes involved mixed-acid fermentation.

#### Simulations

##### Software

Optflux (Rocha et al., 2010), an open source software developed for manipulating metabolic models, was used as the framework for all simulations. Among other functions, this software provides tools for phenotype simulation such as Flux Balance Analysis (FBA) (Varma and Palsson, 1994; Orth et al., 2010).

##### Mutants

The repression of transcription of the *pfl* genes was simulated by limiting the maximum flux through the reactions associated with these genes, to a fraction of the flux in the reference flux distribution (RFD). The RFD can be obtained by performing unconstrained simulations, for different environmental and genetic conditions. To assess anaerobic growth with study 1, two RFDs were determined: the first maximizing biomass with the defined conditions (RFD A.1) and the second by setting the specific growth rate to 0.78 h^−1^ (RFD A.2). The metabolism in different carbon sources involved determining one RFD for each. In this case, all RFDs were determined by maximizing biomass with the same medium, except for the carbon source. RFDs B.1, B.2, B.3, and B.4 refer to glucose, galactose, *N*-acetyl-D-glucosamine, and mannose, respectively.

Hence, for study 1, as *pfl* genes (spr0415 and spr0232) control the flux between pyruvate and acetyl-CoA, simulations using glucose as carbon source were performed by varying the levels of *pfl* between 0 and 100% (deactivated to fully active) of the respective RFDs, with 10% intervals. These simulations allowed assessing the behavior of the model, regarding the level of repression of the mixed-acid genes. Moreover, due to inhibition by CcpA (Schumacher et al., 2004; Carvalho et al., 2011; Fleming et al., 2015; Paixão et al., 2015b) genes *ackA* (spr1854), *adh* (spr1866), and *pta* (spr1007) were severely restricted under anaerobic conditions, as shown in study 1, and were therefore set to 10% of the RDFs while *lctO* (spr0627) was reduced to 0%.

Likewise, for study 2, the flux of *pfl* genes (spr0415 and spr0232), *adh* (spr1866), *pta* (spr1007), and *lctO* (spr0627) were restricted to zero, to simulate growth with glucose under aerobic conditions, as described in Carvalho (2012).

Regarding study 3, simulations for glucose, mannose, and *N*-acetyl-D-glucosamine were performed by implementing the same limitations as for anaerobic conditions, as these genes are controlled by the regulator CcpA, which is active in the presence of these carbon sources (Schumacher et al., 2004; Carvalho et al., 2011; Fleming et al., 2015; Paixão et al., 2015b). For galactose, only flux through reactions associated with the *pfl* genes was varied. The behavior of the bacterium in the different carbon sources was assessed by calculating the by-product formation profiles, which are determined by calculating the proportion of the flux of each by-product, relative to the highest by-product production rate. These profiles allow assessing which mutants, namely the level of repression of the *pfl* genes, better fit the experimental data.

The different mutants used for each study are summarized in Table 1.

**Table 1:** Genetic conditions used in this study.

	Study 1	Study 2	Study 3			
	Anaerobic	Aerobic	Glucose	Mannose	*N*-acetyl-D-glucosamine	Galactose
*adh*	10%	0%	10%	10%	10%	100%
*pta*	10%	0%	10%	10%	10%	100%
*lctO*	0%	0%	0%	0%	0%	100%
*ackA*	10%	100%	10%	10%	10%	100%
*pfl* (spr0415 and spr0232)	Varied from 0 to 100%	0%	Varied from 0 to 100%	Varied from 0 to 100%	Varied from 0 to 100%	Varied from 0 to 100%
References	Carvalho et al., 2013		Paixão et al., 2015a			

*Anaerobic and aerobic simulations were obtained with CDM plus glucose (studies 1 and 2) and carbon sources were simulated in anaerobiosis with CDM (study 3).*

##### Flux variability analysis

The quantitative evaluation of the new model was performed using flux variability analysis (FVA) (Mahadevan and Schilling, 2003) comparing the results of the simulations to data retrieved from previous publications (Carvalho et al., 2013; Paixão et al., 2015b). This analysis included setting the specific growth rate to, at least, 99.9% of the specific growth rate obtained with FBA in the respective RFD.

##### Environmental conditions

The CDM composition utilized by Carvalho et al. (2013) was used to establish the first set of constraints when performing simulations. The rates of consumption and production of metabolites were calculated according previously described methods (Sauer et al., 1999) with data retrieved from Carvalho et al. (2013) study and used to formulate an abstraction of the *in silico* environmental conditions and by-product secretion. The consumption rates of all components of the environmental conditions for all studies were assumed to be the same as the ones calculated with anaerobic growth experimental data, except for the carbon sources and oxygen.

Although *GSM* models simulate steady-state conditions, the experiments considered in this work were performed in batch conditions. Thus, only the exponential growth phase was considered.

In batch cultures, the specific growth rates (μ) can be determined as the coefficient of the log-linear regression of the biomass concentration versus time, whereas the specific rate of substrate consumption (*q*_S_) and the specific rate of product formation (*q*_p_) are the coefficient of the linear regression of substrate [S] or product [P] versus biomass over specific growth rate *X*/μ. This relationship is linear when μ and *q*_S_ are constant (Sauer et al., 1999). For instance, the *q*_S_ for a substrate, e.g., glucose or L-alanine, was calculated as the coefficient of the linear regression of the change in *S* (Δ*S*) against biomass *X* divided by the specific growth rate μ, which is approximately *q*_S_ ≈ $\frac{\Delta \text{S}}{\Delta X/\mu }$.

In study 3, the carbon source consumption rates (*q*_CS_) were calculated using the linear regression approach, using data from the study performed by Paixão et al. (2015b). However, the glucose consumption rates (*q*_Glc_) for studies 1 and 2, summarized in Table 2, were more directly obtained from the data provided by Carvalho et al. (2013). Specifically, the glucose consumption rates were calculated taking into account the biomass yield and growth rates reported by Carvalho et al. (2013). In these cases, the *q*_Glc_ was obtained by dividing the growth rate by the biomass yield and converting it to mmol, according to the following expression: *q*_Glc_ ≈ $\frac{\mu }{{\text{Yield}}_{\text{biomass}}}$ × 1000.

**Table 2:** Environmental conditions studied in this work.

	Study 1	Study 2	Study 3			
Carbon source (mmol g^−1^ h^−1^)	Glucose	Glucose	Glucose	Mannose	*N*-acetyl-D-glucosamine	Galactose
*q*_Glc_	31.71	21.02 (31.71)	34.09	22.65	35.86	32.52
O_2_	0	Unconstrained	0	0	0	0

The final concentrations of the products (namely lactate, acetate, ethanol, and formate) were used to calculate the respective rates.

Anaerobic growth (study 1) was simulated restricting O_2_ flux to zero, whereas for aerobic growth (study 2), oxygen was left unbounded. Finally, the carbon sources assessment (study 3) was performed in anaerobic conditions.

##### Influence of the availability of exogenous amino acids on growth

The CDM composition previously described (Härtel et al., 2012) was used for the analysis of the influence of the availability of exogenous amino acids on growth. Here, each amino acid was removed one at a time from the medium and simulations.

##### Gene essentiality

The experiments reported in study 5 use complex media, thus preventing the determination of the complete list of nutrients. Nevertheless, the CDM by Carvalho (2012) and Carvalho et al. (2013) was used for the *in silico* gene essentiality assessment, as genes considered essential in rich media should also be essential in CDM (Härtel et al., 2012). OptFlux provides a tool that allows determining critical genes automatically, by performing individual gene knockouts and simulating growth.

##### Assessment of model predictions

The simulation results were compared with the data kindly provided by Carvalho et al. (2013) for studies 1 and 2, and by Paixão et al. (2015a) for study 3.

As per study 1, in the absence of oxygen (anaerobic growth) two assessments were performed. In the first, the specific growth rate was maximized and in the second, it was fixed at 0.78 h^−1^, as reported in Carvalho et al. (2013). Both assessments involved determining the maximal and minimal FVAs of lactate, formate, and ethanol for the incremental underexpression of the *pfl* genes, from 0 to 100% (Tables 1, 2, study 1).

Regarding study 2, for aerobic growth (Tables 1, 2, study 2), oxygen was left unconstrained and the FVA of acetate, lactate, and H_2_O_2_ was simulated. Specific growth rate was fixed at 1.07 h^−1^, as reported in Carvalho et al. (2013).

Finally, for study 3, the biomass-specific growth rate and end-products’ fluxes retrieved from model simulations were compared to the experimental data for the different carbon sources. Study 3 (Tables 1, 2) was performed in the absence of oxygen and subject to the genetic restrictions described in Table 1. Such analyses allow determining which level of under-expression of the *pfl* genes allows attaining a fermentation profile similar to the one observed *in vivo*. The Euclidean distance, calculated by determining the squared root of the sum of the squared difference of each element, between the experimental and the simulation’s profiles was determined, according to the following equation:

$${\text{score}}_{\text{profile}}=\sqrt{\sum_{e=1}^{4}{\left(p_{e}^{\text{sim}}-p_{e}^{\text{exp}}\right)}^{2}},$$

Where *e* is one of the four products (lactate, formate, acetate, and ethanol) and *p_e_* is the relative production rate, regarding the product with the largest flux, in either the simulation or experimental data. The best *pfl* under-expression level was determined by the lowest distance to the experimental data.

As, no experimental data for *S. pneumoniae* R6 exists regarding essential amino acids for growth, study 4, in Härtel et al. (2012), was used as the basis to verify flux distributions across the central carbon metabolism, as well as predicting amino acids for which *S. pneumoniae* R6 is auxotrophic.

The comparison of data from study 5 with results obtained from OptFlux simulations was processed as explained below.

For all genes considered essential using OptFlux with a match in OGEE, the reason(s) for essentiality was(were) sought in literature and biological data, and the gene was annotated as essential under those experimental conditions. For cases in which a gene identified as essential by OptFlux was considered non-essential by the OGEE, a first analysis that relied on verifying if the products of the associated reaction were required for biomass production and how many reactions were able to synthesize these products was performed. If the products were required for biomass production and all reactions that led to their production were dependent on that single gene then the gene was considered essential.

For cases in which genes were classified as essential by OGEE but not OptFlux, a first analysis relied on verifying if the reaction products were required for growth. Next, a search for multiple genes assigned the same function was performed. Whenever more than one gene was annotated with the same metabolic capability, a search across several databases and literature was carried out to determine if the annotation was correct. If all genes were correctly annotated, then GPR rules were analyzed. This step relied on analyzing biological data, as well as verifying orthologous genes from KEGG BRITE (Tanabe and Kanehisa, 2012). If GPR rules were correct, then a last analysis was performed. This analysis consisted in verifying if the *in silico* experimental conditions provided the compounds required for biomass production, rendering the reactions that led to their production non-essential. In this case, the simulation constraints were changed (usually by removing the compound from the medium) to ascertain the need for the protein encoding gene(s). If under these new conditions, growth was inhibited, then the gene was classified as probably essential under specific conditions, otherwise it was labeled as probably non-essential.

#### Model Curation

Whenever *in silico* results did not match experimental data, model curation was performed. *Merlin*’s user-friendly graphical user interfaces allow performing re-annotations, correction of reactions’ directionality, and inclusion/exclusion of reactions from the model, as well as exporting the final model in Systems Biology Markup Language (SBML) (Hucka et al., 2003) format.

##### Gap filling

The gap filling process relied on the analysis of the gaps highlighted by a feature available in *merlin*. This tool analyses the connectivity of all metabolites available in the model and determines which ones are either only products or reactants. Then, *merlin* identifies reactions in which these metabolites participate and highlights them. These reactions were analyzed against MetaCyc and KEGG pathways. This process involved identifying reactions in the vicinity of blocked reactions containing dead-end metabolites. Then, enzymes promoting the neighboring reactions were sought in *merlin*’s annotation to identify missing or misannotations. Also, literature on the main pathways in which blocked reactions participate was studied to assess any particularities of the organism, such as auxotrophies.

## Results and Discussion

### Genome Annotation

As stated in the section “Materials and Methods”, a phylogenetic tree (Supplementary Figure S1) was developed to assist during the re-annotation procedure. Analysis of the results demonstrated that all *Streptococcus* species were closer to *S. pneumoniae* R6 than *L. lactis* and *Bacillus subtilis*. However, when no information for streptococci was found, specifically regarding reference strain *S. pneumoniae* D39, searches were first performed using homologous genes from *L. lactis* and secondly from *B. subtilis.* In addition to their close relatedness to *S. pneumoniae* R6, the metabolism of these species has been well described and both have manually curated *GSM* models available.

Re-annotation of the genome identified 1242 metabolic genes out of the 2046 candidates, representing 61% of the whole genome. Several reasons, such as the removal of pseudo and truncated genes, blocked reactions and corresponding encoding genes, removal of genes whose function could not be fully determined (i.e., incomplete EC number), manual curation of both annotation and model (which included actions such as the removal of reactions related to DNA and RNA processes), decreased this number down to the final 372 (18.2%) genes that exist in the model. Examples of outcomes during the re-annotation phase, using the re-annotation workflow, are shown in Supplementary Table S2. The complete list of genes reviewed using this workflow is available in Supplementary Table S3.

Several annotation scenarios were faced, as seen in Supplementary Table S2 of the Supplementary Material. For instance, Spr0009 was classified as encoding a hypothetical protein by both KEGG and the Universal Protein Resource Knowledgebase (UniProtKB) (UniProt Consortium, 2010), but *merlin* was able to classify it as a beta-lactamase encoding gene. The analysis of the protein sequence using CDD suggested that it belongs to the beta-lactamase superfamily, which led to the acceptance of the initial annotation and assignment of the respective EC number.

Gene annotations in which *merlin*’s assignment matched the information retrieved from reviewed genes in UniProt can be regarded with higher confidence. In these cases, information was often replicated throughout the remaining databases, such as the case of gene Spr0021.

The annotation of genes with incomplete EC number, such as the cases of Spr0022 and Spr0064, required an in-depth analysis. Databases such as KEGG, UniProtKB, and CDD were consulted. In the case of Spr0064, the CDD classified the protein as a sugar isomerase. A search of the protein name assigned by BRENDA returned the EC number 5.3.1.26 which also belonged to the sugar isomerase superfamily. Due to the absence of literature support and information on other databases, the gene was annotated with this EC number and assigned the Label E. Regarding gene Spr0022, KEGG assigned it EC number 3.5.4.33 [tRNA(adenine34) deaminase], UniProtKB annotated the product as a hypothetical protein while BioCyc assigned the EC number 3.5.4.5 (cytidine deaminase) to the gene product. Analysis of the protein sequence on CDD revealed that the gene encoded a protein of the cytidine/deoxycytidilate deaminase superfamily and BLAST on UniProtKB revealed similarity to a gene that encodes a cytidine deaminase protein and search of this protein on BRENDA returned the EC number 3.5.4.5. Thus, this gene was assigned the Label E. Labels A, B, and C account for over 86% of the annotation, with approximately 33, 34, and 20% of the classifications, respectively.

There were cases in which genes assigned with incomplete EC numbers (e.g., spr0068) were ultimately annotated with complete ones. Despite the classification as hypothetical protein by KEGG and UniProtKB, the analysis of the conserved domains as well as the availability of complete EC numbers that matched protein function by *merlin* increased reliability of the assigned function (uridine phosphorylase in this example).

Finally, a list of genes annotated as transporters by *merlin*’s TRIAGE is available in Supplementary Table S4.

### Biomass Composition

The biomass macromolecular composition, mostly adapted from *i*AO358, is presented in Table 3. The detailed biomass composition, in terms of compounds, is available in Supplementary Table S5. The proportion of protein in the cell (0.59) and the conversion factor from OD measured at 620 nm (OD620) to grams (which is 1 OD620 to 0.39 g_DW_) was provided in a personal communication by Mafalda Cavaleiro from Rute Neves lab. The cell wall contents were calculated from previous publications (Bui et al., 2012), according to which 1 L of culture contains ∼40–60 mg of cell wall. The cells used in such assays were harvested at an OD620 of 0.5, which can then be converted to 0.195 g_DW_/L. Hence, the average cell wall contents (50 mg CW/L) represent about 0.26 g of cell wall per gram of biomass.

**Table 3:** Biomass macromolecular composition of the model *i*DS372 from *Streptococcus pneumoniae* R6 and comparison with biomass from iAO358 *Lactococcus lactis* ssp. Lactis IL1403.

Biomass component	*Lactococcus lactis* ssp. Lactis IL1403	*Streptococcus pneumoniae* R6
	g gDW^−1^	g gDW^−1^
Protein	0.46	0.59
DNA	0.023	0.0087
RNA	0.107	0.0403
Lipoteichoic acid	0.08	0.0302
Lipids	0.034	0.0128
Peptidoglycan	0.118	–
Polysaccharide	0.12	–
Cell wall	–	0.2600
Cofactors and others	–	0.0580
Total	0.942	1
Total (wo/polysaccharide)	0.822	–

The remaining macromolecular relative contents were obtained from *i*AO358. As strain R6 lacks capsule, the capsular polysaccharide contents in *i*AO358 were distributed by the other macromolecules, maintaining the proportions for each component.

As mentioned previously, proteins, DNA, and RNA composition were determined using the e-BiomassX tool, available in *merlin* (Supplementary Table S5).

Cell wall composition was determined according to a previous study (Bui et al., 2012), which suggests that the teichoic acid represents 40**–**50% of the cell wall dry weight, and Fischer (1997) provided a detailed description of the teichoic acid composition. The peptidoglycan composition was reconciled between the KEGG reactions assigned through similarity by *merlin* to its biosynthesis pathway and the study by Mosser and Tomasz (1970), which reports this molecule elemental composition (Supplementary Table S6).

The lipoteichoic acid (Supplementary Table S7) composition was determined from the literature, namely the study by Behr et al. (1992). This study also allowed determining the average fatty acyl molecule (Supplementary Table S8), which is a precursor to all lipids.

The average lipid composition (Supplementary Table S9) was inferred from the *L. lactis***’** model. However, the lysophosphatidylglycerol molecule was removed from the lipid composition since this molecule was not present in the model and its contents were distributed among the other molecules.

Although not available in the *L. lactis* model, a placeholder for the cofactors and other molecules was added to this model. Usually, glutathione is included in the cofactors pool. However, a study (Potter et al., 2012) showed that *S. pneumoniae* lacks genes for biosynthesizing glutathione, thus extracellular glutathione is imported by an ABC transporter substrate-binding protein GshT and is used as a cysteine source when defending against oxidative stress and metal ion toxicity. As the defined medium used by Carvalho et al. (2013) does not include glutathione, this was not included in the biomass cofactors.

The growth associated energy requirements, calculated from the work of Carvalho et al. (2013), indicate that generating 1 g of biomass requires 63 mmol_*ATP*_ h^−1^.

### Metabolic Model

The complete list of reactions in the *i*DS372 model can be found in Supplementary Table S10. The metabolic model developed in this work is composed of 462 reactions + 67 drain reactions and 372 genes. Regarding the reactions, 75 are associated with transport phenomena and 20 were added manually to the model to fill gaps in the network, though in most cases the justification was found in literature and other databases, according to the “notes” column in Supplementary Table S10.

As illustrated in Table 4, although the ratio of the number of genes in the model over the total number of genes in the organism (18 %) is similar to that of published models for closely related organisms such as *L. lactis* (Oliveira et al., 2005), *B. subtilis* (Oh et al., 2007), and *Streptococcus thermophilus* (Pastink et al., 2009), this model requires less reactions.

**Table 4:** Comparison of *i*DS372 model from *S. pneumoniae* strain R6 with several other published models.

Organism	Genes	Genes in model	Gene ratio	Metabolites	Reactions	Compartments	References
*Streptococcus pneumoniae* R6	2043	372	0.182	355(67)	462	2 (c, e)	This work
*Streptococcus thermophilus* LMG18311	1889	429	0.227	550	522	2 (c, e)	Pastink et al., 2009
*Lactococcus lactis* ssp. Lactis IL1403	2310	358	0.155	422	621	2 (c, e)	Oliveira et al., 2005
*Bacillus subtilis* 168	4114	844	0.205	988	1020	2 (c, e)	Oh et al., 2007

*c, cytosol; e, extracellular space.*

This model has 409 GPR associations, listed in Supplementary Table S10 of the Supplementary Material.

The GSM model (iDS372) in the SBML version 3 format is included in the Supplementary Material (iDS372.xml).

### Model Validation

#### Environmental Conditions

The validation of this model involved using an abstraction of the CDM, developed with the aim of providing a high pneumococcal growth yield, by Carvalho et al. (2013).

The results of the calculations performed according to Sauer et al. (1999), to convert the consumption rates under CDM into *in silico* environmental conditions, are shown in Supplementary Table S11.

For instance, after the exponential growth phase (μ = 0.78 h^−1^), Δ_X_ = 1.27 g_Biomass_ L^−1^ and Δ_l–alanine_ = −0.24 g L^−1^. Thus, according to the calculations for determining the substrates specific consumption rates performed using linear regressions, the *q*_l–alanine_ = 1.66 mmol_l–alanine_ g_Biomass_^−1^ h^−1^. The linear regression approach was used to determine the *q*_S_ for every metabolite in that table, except glucose and oxygen. Whereas the latter was left unbounded in aerobic conditions (and bounded to zero in anaerobic conditions), the former was retrieved from Carvalho et al. (2013) using the expression presented in the section “Environmental conditions”: *q*_Glucose_ = μ ×*Y*_X/S_^−1^ × 1000 ≈ 31.71 mol_glucose_ ${\text{g}}_{\text{Biomass}}^{-1}$ h^−1^ (μ = 0.78 h^−1^, *Y*_X/S_ = 24.6 g_Biomass_ ${\text{mol}}_{\text{glucose}}^{-1}$ and assuming the maintenance negligible).

Likewise, an abstraction of the medium used by Härtel et al. (2012) was used for the evaluation of amino acid requirement (Supplementary Table S12). As this medium was used in our experiments for essentiality analysis only (qualitative assessment), the value of the consumption rate of each metabolite was not so relevant. Thus, in this case the substrates’ specific consumption rates were the same as in Carvalho et al. (2013) for the common metabolites, whereas all the others were set to −10 mmol_glucose_ ${\text{g}}_{\text{Biomass}}^{-1}$ h^−1^.

The growth dependency of *S. pneumoniae* on exogenous choline is well known, as this compound is used to assemble this organism’s unusual teichoic acids (Tomasz, 1967). Previous experiments have shown that doubling the choline concentration increased the biomass yield by 30% (Carvalho et al., 2013). Therefore, to avoid artifacts in simulations caused by the limitation of choline (data not shown), the choline uptake rate was left unconstrained in the environmental conditions.

#### Maintenance ATP

Although using a flux of 1 mmol_ATP_ ${\text{g}}_{\text{Bio}}^{-1}$ h^−1^ as the initial estimate, a range of FVA, between 1 and 10, was assessed to evaluate the best fit to the model growth rate. This evaluation was performed both in anaerobic and aerobic conditions, with the *pfl* gene completely deactivated, as in Carvalho et al. (2013) no formate was formed in both conditions by *S. pneumoniae* R6. As shown in Supplementary Table S13, the selected value for the anaerobic maintenance ATP flux was 6.5 mmol_ATP_ ${\text{g}}_{\text{Bio}}^{-1}$ h^−1^, as this flux provides an *in silico* growth rate similar to the experimental data (0.78 h^−1^). Likewise, the aerobic maintenance ATP flux was set to 4.5 mmol_ATP_ ${\text{g}}_{\text{Bio}}^{-1}$ h^−1^. The remaining simulations, shown in the present work, were performed considering these estimates.

#### Simulations’ Assessment

**FIGURE 2:**
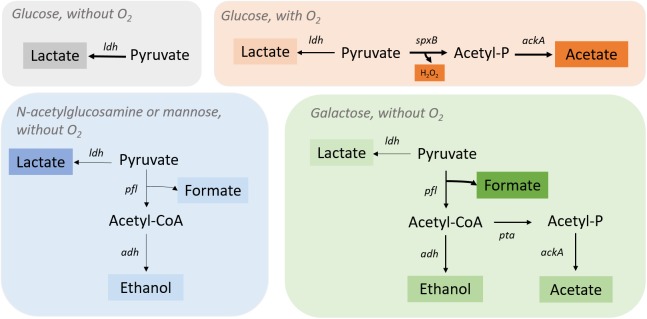
*Streptococcus pneumoniae* R6 pyruvate metabolism under different environmental conditions. Pyruvate is fully converted into lactate by lactate dehydrogenase (Ldh) when glucose is present, and oxygen is absent. In the presence of oxygen, *S. pneumoniae* R6 switches to a heterofermentative profile by activating the *spxB* (pyruvate oxidase) gene to produce acetate and H_2_O_2_ together with lactate (in minor quantities). In the presence of *n*-acetylglucosamine or mannose (without O_2_), the *ldh* gene is fully activate leading to the production of lactate as major fermentation product and *pfl* (pyruvate formate-lyase) genes are only partially activated, as shown by the minor quantities of acetate, ethanol, and formate. In the presence of galactose, formate is the major product of fermentation revealing a complete activation of the *pfl* genes. Ethanol and acetate are also produced, ethanol by the action of alcohol dehydrogenase (Adh) and acetate from acetyl-CoA production by phosphate acetyltransferase (Pta) and acetate kinase (AckA).

The first validation performed to the *i*DS372 model involved comparing simulation results to the work of Carvalho et al. (2013). Regarding this validation, the model was used to simulate the behavior of the microorganisms under anaerobic and aerobic conditions. A map of the main variants of the central metabolism of *S. pneumoniae* R6 is depicted in Figure 2.

##### Anaerobic growth

It is well known that PFL is an intrinsic part of the mixed-acid fermentation process. After being synthesized, it has to be activated via a PFL-activating enzyme (Melchiorsen et al., 2000; Buis and Broderick, 2005). Also, PFL is highly sensitive to the presence of molecular oxygen and performs better in hypoxic conditions (Yamada et al., 1985; Melchiorsen et al., 2000; Takahashi-Abbe et al., 2003; Buis and Broderick, 2005). Moreover, in D39, PFL is activated post-translationally and glucose oxidation intermediates, such as glyceraldehyde-3-phosphate and dihydroxyacetone phosphate, inhibit the flux through the PFL (Yesilkaya et al., 2009). In this strain, glucose favors homolactic fermentation, since genes associated with mixed-acid fermentation, namely PFL spr0415 (*pfl*) and spr0232 (*pflF*), acetokinase spr1854 (*ackA*), alcohol dehydrogenase spr1866 (*adh*), and phosphate acetyltransferase spr1007 (*pta*) are repressed, and lactate dehydrogenase spr1100 (*ldh*) is activated, which confirms the prominent role of PFL in the by-product flux distribution. As discussed before, in anaerobic conditions using glucose as carbon source, *S. pneumoniae* strain R6 produces mainly lactic acid (93%) together with mixed-acid fermentation products, namely formate, acetate, and ethanol, which account for only 7% for the total of fermentation products as shown in Carvalho et al. (2013).

**FIGURE 3:**
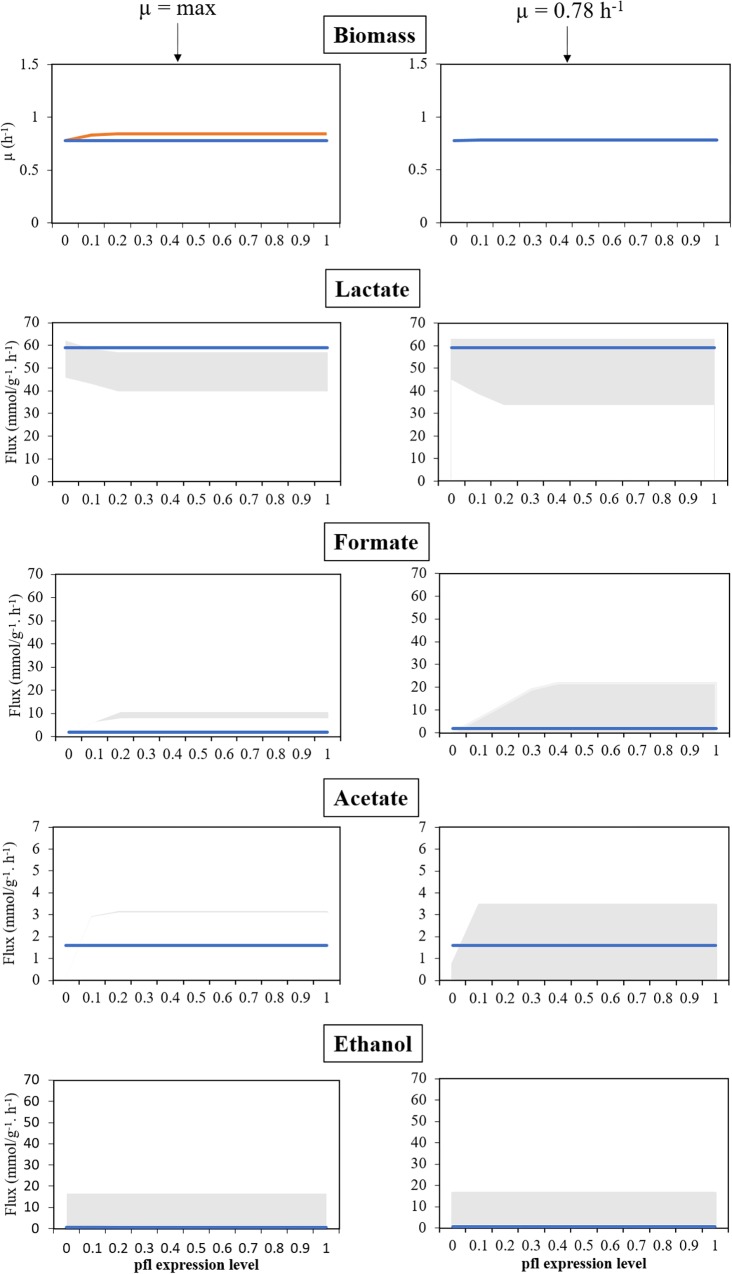
Model assessment in anaerobic conditions, for several levels of expression of the *pfl* genes, *in silico*, against experimental data. Left panel corresponds to a maximization of growth rate as objective function. Right panel corresponds to simulations with fixed maximum growth rate (μ = 0.78 h^−1^). Blue lines represent experimental data, the orange line represents the maximization of the specific growth rate, and the shadowed areas represent the products’ flux variability analysis. All simulations were performed with environmental conditions inferred from Carvalho et al. (2013).

Regarding the simulation using the *S. pneumoniae* R6 model in anaerobic conditions (study 1), FVA was performed for each of the by-products, while simultaneously varying the level of expression of *pfl* between 0 and 100%. This assessment involved restricting the flux of the reactions promoted by PFL to different ratios of the flux obtained in RFDs A.1 and A.2 for such reactions, while simulating the maximization and minimization of the production of each metabolite, and the results of these simulations are shown in Figure 2, 3.

As shown in the left panel of Figure 3, FVA simulations performed with RFD A.1 reveal that the growth rate is positively correlated with the level of activity of the genes involved in PFL synthesis. When genes encoding PFL are completely silenced, the simulated growth rate is similar to the experimentally determined (0.78 h^−1^), which was expected as this rate was used to determine the maintenance ATP requirements. However, in these conditions, the model cannot provide the observed by-products profile, as though silencing the *pfl* genes in the simulation allows generating enough lactate, formate, and acetate cannot be produced in sufficient amounts. When the flux of the reactions promoted by PFL was restricted to 10% of RFD A.1, the growth rate increases to ∼0.83 h^−1^, and the minimum amount of formate produced by this bacterium was 2.86 mmol_formate_ ${\text{g}}_{\text{Biomass}}^{-1}$ h^−1^, more than the one attained experimentally (1.9 mmol_formate_ ${\text{g}}_{\text{Bio}}^{-1}$ h^−1^), not allowing a recapitulation of the by-products profile. However, in this case, the maximum production of lactate in the simulation is identical (58.27 mmol_lactate_ ${\text{g}}_{\text{Bio}}^{-1}$ h^−1^) to the one experimentally determined (58.98 mmol_lactate_ ${\text{g}}_{\text{Biomass}}^{-1}$ h^−1^).

The next set of simulations (right panel of Figure 3) involved performing FVA simulations with RFD A.2, in which the specific growth rate was limited to 0.78 h^−1^. The assessment of the influence of the genes encoding PFL demonstrates that, like before, when these genes are silenced the organism cannot produce enough formate. Instead, it redirects the metabolism to homolactic fermentation. Increasing PFL activity induces a shift from lactate to mixed acid fermentation products, namely formate that was found in minor amounts in the experimental conditions, which indicates that the *pfl* was not completely silenced. Restricting growth allows mimicking the experimental results for all levels of expression of genes encoding the *pfl*, except for the knockout (0%) as this restriction impairs the production of formate and acetate when compared to the experimental data. The experimental by-products profile can be reached in all simulations (except the case described before), when performing FVA analyses. Nevertheless, such profile is more robust when the expression of genes encoding PFL is closer to 0, as the lower the expression results in a lower the production of formate, as depicted in Figure 3. Therefore, the model is in good agreement with experimental data.

##### Aerobic growth

In the presence of oxygen and having glucose as carbon source, *S. pneumoniae* changes from producing lactate to producing acetate and H_2_O_2_ due to a highly active pyruvate oxidase activity. Even more, lack of formate and ethanol denotes the complete inactivity of PFL under these conditions (Carvalho et al., 2013). Genome comparison of strains D39 and R6 shows 71 single base-pair changes, 6 deletions, 4 insertions, and loss of the pDP1 plasmid (Lanie et al., 2007). In agreement with these relatively minor genetic differences, the metabolic and physiological behavior of these two strains is considerably similar. The main difference between these two strains is the presence of a capsule in the parental D39 strain. Besides, in comparison to D39, *S. pneumoniae* strain R6 has a more active pyruvate oxidase activity (Belanger et al., 2004; Ramos-Montañez et al., 2008), which increases the production of acetate and H_2_O_2_ in the presence of oxygen (Carvalho et al., 2013). It is well known that *Streptococcus* species are highly sensitive to the production of H_2_O_2_, as the minimal inhibitory concentration is only 1 mM of H_2_O_2_ (Pericone et al., 2003). This phenomenon is explained by the fact that these strains are catalase negative (Hoskins et al., 2001; Lanie et al., 2007). Therefore, in the presence of oxygen, the “faster” metabolism in strain R6 provides a transitory advantage regarding strain D39, as a faster H_2_O_2_ accumulation will compromise its survival (Carvalho et al., 2013).

Simulations performed under aerobic environmental conditions (study 2) were rather different from the ones performed under the absence of oxygen, as shown in Figure 2, 4. In this case, experimental data from Carvalho et al. (2013) reports a growth rate of 1.07 h^−1^ and a biomass yield of 50.9 g_Biomass_ ${\text{mol}}_{\text{Glucose}}^{-1}$, which corresponds to a glucose consumption rate of 21.02 mmol_glucose_ h^−1^ g^−1^. For comparison purposes, the same *q*_Glc_ (31.71 mmol_glucose_ h^−1^ g^−1^) used for anaerobic simulations was also tested for aerobic growth.

**FIGURE 4:**
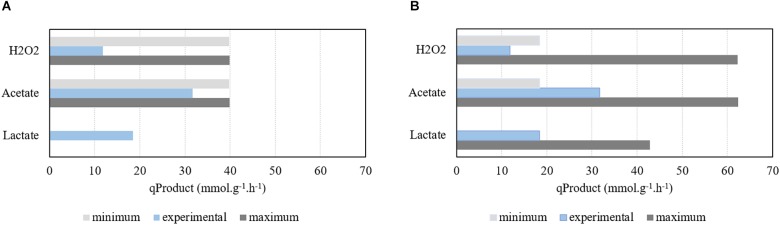
Assessment of the model simulations performed under aerobic conditions for different glucose uptake rates (qGlucose) and comparison with experimental results. **(A)** Production of H_2_O_2_, acetate, and lactate using a qGlucose of 21.02 mmol g^−1^ h^−1^. **(B)** Production of H_2_O_2_, acetate, and lactate using a qGlucose of 31.71 mmol g^−1^ h^−1^. Growth rate was limited to 1.07 h^−1^.

Hence, simulations were performed with the specific growth rate limited to 1.07 h^−1^, while deactivating genes encoding the PFL enzyme and maximizing/minimizing acetate, H_2_O_2_, and lactate production, as described in the section “Materials and Methods”. Results show that, in these conditions, production of acetate and H_2_O_2_ is mandatory, whereas lactate’s FVA shows that only trace amounts of this metabolite can be produced (Figure 4A). In fact, the minimum production of both acetate and H_2_O_2_ are excessive when compared with data calculated from the information provided by Neves and coworkers. A higher glucose consumption rate was used for comparison purposes. In this case, Figure 4B shows that the model is able to encompass the experimental data results profile. Moreover, according to the simulation, in aerobic conditions increasing the carbon source uptake rate will allow to decrease the production of acetate. The acetate kinase provides two molecules of ATP to the metabolism. However, increasing the carbon source uptake rate allows the pneumococcus to obtain ATP through the pyruvate kinase. Therefore, while fixing the maximum growth rate, increasing the carbon source uptake rate allows obtaining more ATP through glycolysis and consequently to decrease the minimum requirements of acetate production.

The experimental data assessment, calculated as described above, is impaired by the short period of time in which the exponential growth phase took place that limited to two the number of experimental time points used to determine the product formation rates. In fact, quantification issues were detected in the experimental data, as one molecule of H_2_O_2_ should be produced per each molecule of acetate. This is observed in the model simulation results but not experimentally, in which the flux of H_2_O_2_ is much lower than the one of acetate, due to the spontaneous decay of H_2_O_2_ into water and molecular oxygen.

Nevertheless, the model is able to perform viable simulations under aerobic conditions producing acetate, H_2_O_2_, and lactate as shown experimentally.

##### Carbon sources

The iDS372 model is able to simulate growth in all tested substrates (study 3) under anaerobic conditions. As seen before, it is possible to simulate different types of fermentation by varying *pfl* expression from 0 to 100%, which involves restricting the flux through the reactions catalyzed by these genes in the respective RFDs (see Supplementary Table S14A for full simulations). The Euclidean distance, calculated as described before, allowed inferring which percentage of *pfl* under-expression should be set in the model, for each carbon source, to mimic the experimental data of Paixão et al. (2015b) for the parent strain D39. As shown in Table 5, glucose and *N*-acetyl-D-glucosamine products have a fermentation profile closer to an expression of 0% *pfl* of the RFDs B.1 and B.3, respectively. Whereas the mannose fermentation profile is better simulated when restricting the flux of the reactions promoted by PFL to 10% of the flux obtained in RFD B.4 for such reactions, when compared to the experimental data profile. Regarding galactose, the *pfl* under expression should be set to 90%, in the model, of RFD B.2, which is consistent with a higher mixed acid fermentation activity, while guaranteeing that lactate is also produced. Supplementary Table S14 shows the results of the calculation of all distances.

**Table 5:** Growth and by product analysis with the *i*DS372 model, using different (optimized) levels of *pfl* expression for the four different carbon sources tested (glucose, galactose, *N*-acetyl-D-glucosamine, and mannose).

Carbon source	Uptake (mmol g^−1^ h^−1^)	*pfl* (%)	μ (h^−1^)		Lactate (mmol g^−1^ h^−1^)		Formate (mmol g^−1^ h^−1^)		Acetate (mmol g^−1^ h^−1^)		Ethanol (mmol g^−1^ h^−1^)	
			sim	exp	sim	exp	sim	exp	sim	exp	sim	exp
Glucose	34.09	0	0.85	0.82	66.70	57.52	0	0	0	0.9	0	0.11
Galactose	22.65	90	0.82	0.47	4.10	3.78	41.81	28.67	20.61	14.53	19.15	13.93
*N*-acetyl-D-glucosamine	35.86	0	0.89	0.53	70.00	61.37	0	2.03	35.86	1.15	0	0.6
Mannose	32.52	10	0.85	0.41	58.77	49.02	6.80	5.71	3.04	3.0	1.63	2.98

*sim, simulation; exp, experimental.*

As expected, the results for galactose show the prevalence of mixed acid fermentation, while mainly the homolactic fermentation profile is exhibited for glucose and an increase in the fermentation behavior is seen in mannose, all as described in Paixão et al. (2015b). *N*-acetyl-D-glucosamine simulation results are the farthest from the experimental results from all tested carbon sources, with a seemingly anomalous production of acetate. This result is explained by the fact that acetate is a by-product of *N*-acetyl-D-glucosamine degradation. In the model, genes spr0668 and spr1528 encoding phosphoenolpyruvate-dependent phosphotransferase sugar-specific systems transport this compound into the cell converting it to *N*-acetyl-D-glucosamine-6-phosphate and releasing pyruvate. Then, gene spr1867 encoding an *N*-acetyl-D-glucosamine-6-phosphate amidohydrolase converts the phosphorylated compound into acetate and D-glucosamine-6-phosphate, which explains the behavior shared between this carbon source and glucose. Therefore, study 3 assessment allowed determining the level of activation of *pfl* genes in different carbon sources, which until date has not been described.

##### Influence of the availability of exogenous amino acids on organism growth

The results obtained by simulating the environmental conditions used in Härtel et al. (2012) (study 4) with the *i*DS372 model are shown in Supplementary Table S11 of the Supplementary Material.

Overall, mimicking their experiment *in silico* using this model yields results showing a high degree of similarity (80%) to the results obtained by Härtel et al. (2012). Our model confirmed that *S. pneumoniae* R6 although auxotrophic for L-arginine, L-cysteine, glycine, L-histidine, and L-valine contains all reactions required for *de novo* biosynthesis of this amino acids. Likewise, the unconventional pathway for the *de novo* biosynthesis of serine suggested by their study was also confirmed. The mismatching results were in respect to the amino acids isoleucine, leucine, valine, and glutamine. Härtel suggested that *S. pneumoniae* D39 was auxotrophic for these amino acids, as this strain did not grow in their absence, but *in silico* results performed in this study could not confirm this. The annotation of genes involved in the *de novo* synthesis of these amino acids and the lack of additional data to fine tune the pathways or determine how and when these genes are expressed prevent any attempt to further curate these pathways. Hence, these pathways either have regulatory mechanisms preventing the biosynthesis of these amino acids or some of the enzymes involved cannot sustain the flux required for biomass growth. The study from Härtel et al. (2012) also showed that *S. pneumoniae* D39, in the absence of glutamate, proline, and methionine, presented a decreased growth rate. The independent omission of these amino acids from the medium, *in silico*, did not have any impact on the growth rate which was expected as this validation was qualitative and not quantitative. The fact that Härtel et al. (2012) used the parent strain of *S. pneumoniae* R6 could also explain the differences observed in these results.

##### Gene essentiality

As previously stated in the section “Materials and Methods”, study 5 involved performing the gene essentiality analysis with OptFlux’s gene essentiality tool using the anaerobic experimental conditions of Carvalho et al. (2013). A total of 89 genes were identified as essential in our model. The data collected from OGEE database comprised a total of 133 essential genes identified for *S. pneumoniae* strain R6. Out of the 89 essential genes, 67 were not listed as essential by OGEE. Only 50 genes identified as essential by OGEE were present in our model and from these only 23 matched the results obtained by OptFlux’s gene essentiality tool. From this comparison, another nine genes could be considered essential if certain experimental conditions were met such as the removal of some amino acids (i.e., tryptophan or methionine), vitamins (i.e., folate) from the medium composition or certain GPR rules were adjusted. The remaining 27 genes would never be classified as essential in this model due to the nature of stoichiometric models that do not take in account different catalytic activity of isoenzymes.

OptFlux’s results were labeled as the “predicted” and the list of genes obtained from OGEE that exist in *i*DS372 as the “real” results shown in Table 6, to determine the performance of this model, in terms of predicting gene essentiality.

**Table 6:** Confusion matrix and respective performance measure calculations of the iDS372 model in predicting essential genes.

	Exp. positive	Exp. negative
Predicted positive	23	66
Predicted negative	27	256
**Measure**	**Value**	**Derivations**
Sensitivity	0.4600	TPR = TP/(TP + FN)
Specificity	0.7950	SPC = TN/(FP + TN)
Precision	0.2584	PPV = TP/(TP + FP)
Negative predictive value	0.9046	NPV = TN/(TN + FN)
False positive rate	0.2050	FPR = FP/(FP + TN)
False discovery rate	0.7416	FDR = FP/(FP + TP)
False negative rate	0.5400	FNR = FN/(FN + TP)
Accuracy	0.7500	ACC = (TP + TN)/(P + N)

*Genes considered essential in OGEE are defined as “True”, while genes identified as essential by OptFlux’s prediction tool are considered the “Predicted”.*

Overall, the results obtained clearly demonstrate that the model performs well in the discrimination of essential from non-essential genes with a high level of accuracy (75%). The model performs very well in predicting non-essential genes, shown by the high specificity (∼79%) and negative predictive value (∼90%). The prediction of essential genes is, in comparison to the non-essential, lower. This is shown by the relatively low sensitivity (46%) and precision (26%). A possible explanation for this relies on the fact that the genes identified as essential by OptFlux are directly linked to metabolism and influenced by the medium composition (e.g., presence/absence of folate). On the other hand, the essential gene list obtained from OGEE, although generated using a complex medium, includes non-metabolic genes. Hence, differences in the number of essential genes from each approach were expected. For an improved validation of the results, *in vitro* or *in vivo* gene knockout studies should be performed using the defined medium described in Carvalho et al. (2013).

In Table 7, some examples of the results obtained from the gene essentiality study are shown. A complete list of all essential genes predicted by the model, as well as those obtained from OGEE database is available in Supplementary Table S15.

**Table 7:** Examples of the analysis of essential genes presented in metabolic model.

Locus tag	Result	Enzyme function	GPR rules	Notes	Specific conditions
spr0245	Essential	Glutamine-fructose-6-phosphate transaminase (isomerizing)	spr0245	Catalyzes a step reaction that leads to *N*-acetyl-D-glucosamine which is essential for peptidoglycan production.	–
spr0266	Non-essential	Dihydropteroate synthase	spr0266	The enzyme encoded catalyzes a key reaction that leads to folate synthesis. Folate derivatives are essential cofactors in purine, pyrimidine, and amino acid biosynthesis.	Removal of folate from the medium renders this gene essential,
spr1312	Non-essential	Thioredoxin-disulfide reductase	spr1602 or spr1312	Classified as a defense mechanism against oxidative stress and redox regulation of protein function.	Conditions in which each gene is expressed are unknown.
spr0180	Essential	cardiolipin synthetase	spr0180	Produces the necessary cardiolipin for biomass	–

## Conclusion

The main objective of this study was to reconstruct a GSM model for the *S. pneumoniae* R6 strain, capable of predicting essential genes and simulating phenotypic behavior. This is the first manually curated GSM model to be reconstructed for any strain of *S. pneumoniae* and establishes the groundwork for a better understanding of the metabolism of this major pathogen.

A high level of manual curation, based on literature, experimental data, and biological databases, was performed when constructing the network, which should increase the reliability of the model. The prominent role of the PFL regulation in *S. pneumoniae* proposed by Carvalho and colleagues was confirmed in this study. Also, this model was able to replicate *S. pneumoniae*’s behavior under different environmental conditions, including different carbon sources and oxygen availability. Considering the overall results obtained, *i*DS372 can be employed to provide reliable qualitative or quantitative simulations under different experimental conditions.

The medium used by Carvalho (2012) is very rich, as it contains several amino acids; thus, various compounds can be sources of carbon, nitrogen, and sulfur. These circumstances, together with the lack of chemostat data do not allow a full validation of the model. The availability of data with minimal media and chemostat conditions will allow further validation studies.

*i*DS372’s predictions confirmed almost all essential amino acids under the conditions established by Härtel et al. (2012). The fact that *S. pneumoniae* R6 possesses all the genes required to synthesize the amino acids whose results did not match suggests that other factors may regulate their expression in *S. pneumoniae* R6. Further studies on this topic can provide additional information on the differences between R6 and D39.

*i*DS372 performed well on all five studies used to validate its phenotypical predictions to different genetic and environmental conditions.

Finally, the model provided in the SBML level 3 version 2 was able to score over 97% on all consistency tests on the Memote (Lieven et al., 2018) test suite, except for the charge balance, which was not accounted for in this work, and the unbounded flux in default medium.

The lack of experimental data of quantitative nature limits the spectrum of application of this model. The elaboration of new studies using CDM and less rich media clearly defined environmental conditions, and quantification of both substrate consumption as well as by-product formation would yield more information, which could be used to further curate the network and enhance and extend its predicting capabilities.

Considering the natural genetic diversity within the *S. pneumoniae* species, and the fact that *i*DS372 is the first curated GSM model for this species, it will be a pivotal model to study the impact of that genetic diversity in the metabolic capabilities of specific strains with potentially relevant clinical correlations.

## Data Availability

This manuscript contains previously unpublished data. The name of the repository and accession number are not available.

## Author Contributions

OD and IR designed the research and provided guide throughout the investigation. OD, IR, and FP coordinated the project. JS and OD developed and refined the model. OD, JS, MR, and CF performed the model validation and evaluation. All the authors wrote the manuscript, read, and approved the final version of the manuscript.

## Conflict of Interest Statement

The authors declare that the research was conducted in the absence of any commercial or financial relationships that could be construed as a potential conflict of interest.

## Footnotes

1 https://www.ebi.ac.uk/Tools/msa/clustalo/
