# Supplementary material for: iDS372, a Phenotypically Reconciled Model for the Metabolism of Streptococcus pneumoniae Strain R6
Source: Front Microbiol. 2019 Jun 25;10:1283. doi: 10.3389/fmicb.2019.01283 (PMC6603136; doi:10.3389/fmicb.2019.01283)
Supplement: Supplementary file 1 [file Data_Sheet_1.pdf]

## Supplementary Material

### 1 Supplementary Figures

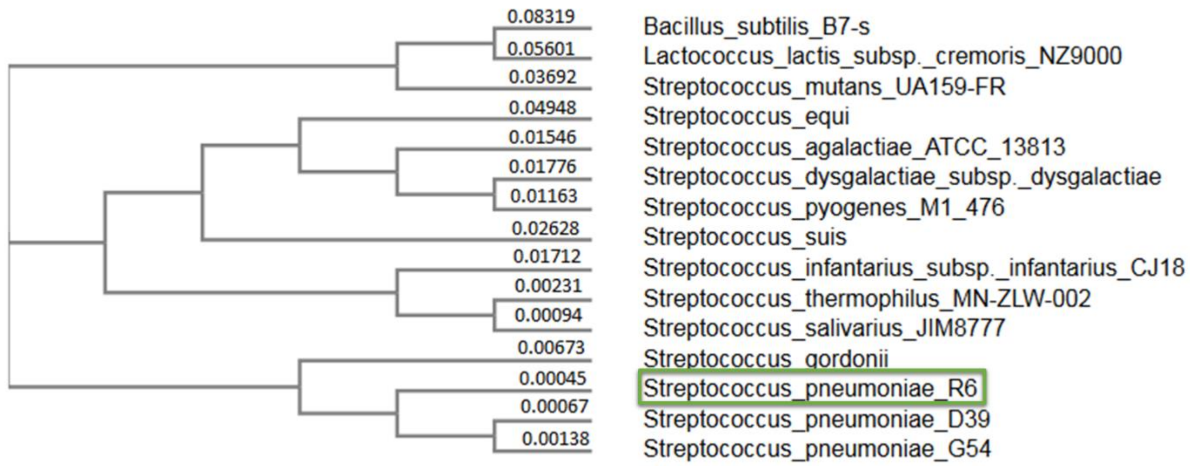


**Supplementary Figure 1.** Phylogenetic tree of Streptococcus pneumoniae relative organisms. This tree was built using EMBL-EBI Clustal OMEGA multiple sequence alignment tool (Sievers et al., 2011). Numbers above each line represent the branch lengths to each node generated automatically by the tool using the Neighbor-joining method (Saitou and Nei, 1987).

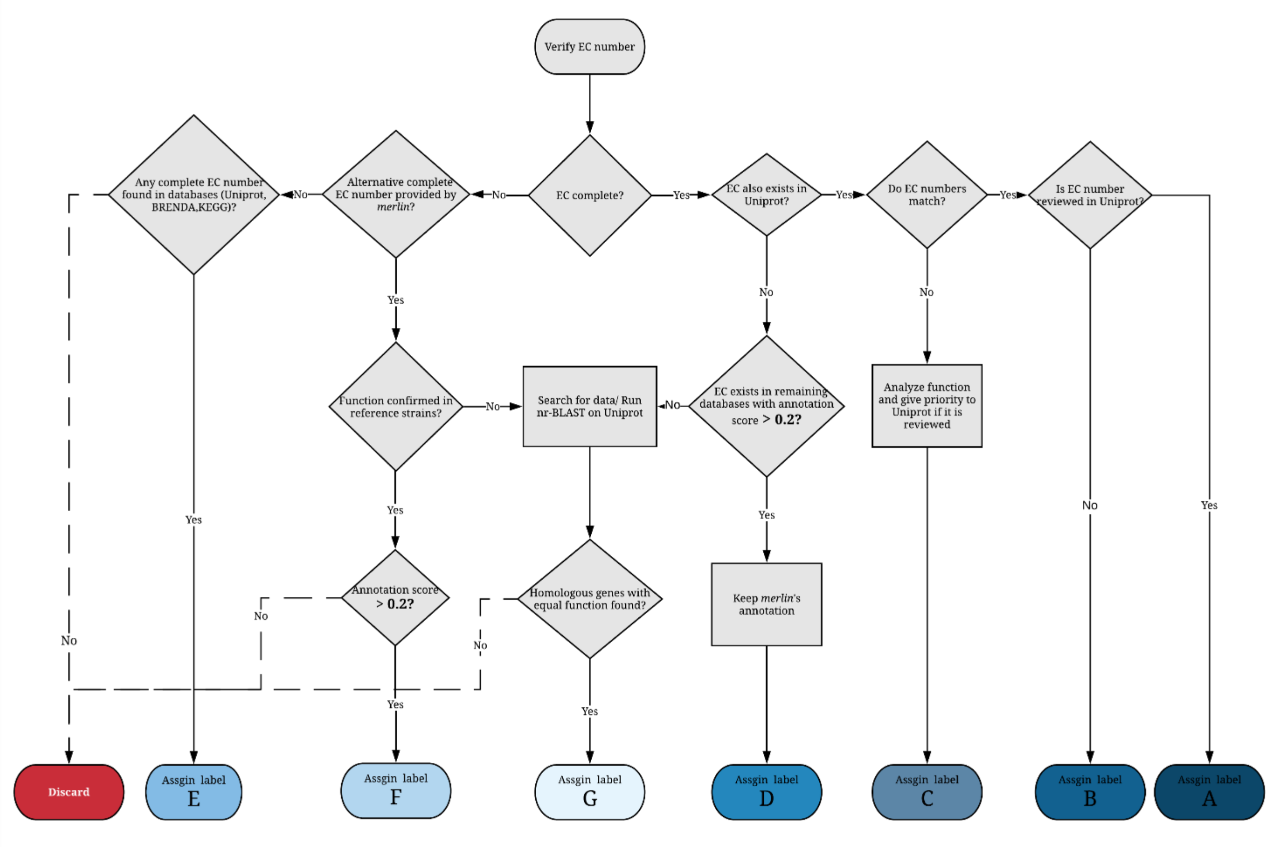


**Supplementary Figure 2.** Re-annotation workflow for model iDS372.
